# The Effect of High-Fat Diet on Intramyocellular Lipid Content in Healthy Adults: A Systematic Review, Meta-Analysis, and Meta-Regression

**DOI:** 10.1016/j.tjnut.2024.02.026

**Published:** 2024-02-26

**Authors:** Jasem Alqallaf, Samuel T Orange, Jamie Matu, Alex Griffiths, Kelsie Johnson, Antonis Stavropoulos-Kalinoglou, Adrian Holliday, Oliver Wilson

**Affiliations:** 1 Carnegie School of Sport, Leeds Beckett University, United Kingdom; 2 School of Biomedical, Nutritional, and Sport Sciences, Faculty of Medical Sciences, Newcastle University, United Kingdom; 3 Newcastle University Centre for Cancer, Newcastle University, United Kingdom; 4 School of Health, Leeds Beckett University, United Kingdom; 5 Research Institute for Sport and Exercise Sciences, Liverpool John Moores University, United Kingdom

**Keywords:** intramuscular triglyceride, lipid droplet, overfeeding, insulin resistance

## Abstract

Fatty acids are stored within the muscle as intramyocellular lipids (IMCL). Some, but not all, studies indicate that following a high-fat diet (HFD), IMCL may accumulate and affect insulin sensitivity. This systematic review and meta-analysis aimed to quantify the effects of an HFD on IMCL. It also explored the potential modifying effects of HFD fat content and duration, IMCL measurement technique, physical activity status, and the associations of IMCL with insulin sensitivity. Five databases were systematically searched for studies that examined the effect of ≥3 d of HFD (>35% daily energy intake from fat) on IMCL content in healthy individuals. Meta-regressions were used to investigate associations of the HFD total fat content, duration, physical activity status, IMCL measurement technique, and insulin sensitivity with IMCL responses. Changes in IMCL content and insulin sensitivity (assessed by hyperinsulinemic-euglycemic clamp) are presented as standardized mean difference (SMD) using a random effects model with 95% confidence intervals (95% CIs). Nineteen studies were included in the systematic review and 16 in the meta-analysis. IMCL content increased following HFD (SMD = 0.63; 95% CI: 0.31, 0.94, *P* = 0.001). IMCL accumulation was not influenced by total fat content (*P* = 0.832) or duration (*P* = 0.844) of HFD, physical activity status (*P* = 0.192), or by the IMCL measurement technique (*P* > 0.05). Insulin sensitivity decreased following HFD (SMD = –0.34; 95% CI: –0.52, –0.16; *P* = 0.003), but this was not related to the increase in IMCL content following HFD (*P* = 0.233). Consumption of an HFD (>35% daily energy intake from fat) for ≥3 d significantly increases IMCL content in healthy individuals regardless of HFD total fat content and duration of physical activity status. All IMCL measurement techniques detected the increased IMCL content following HFD. The dissociation between changes in IMCL and insulin sensitivity suggests that other factors may drive HFD-induced impairments in insulin sensitivity in healthy individuals.

This trial was registered at PROSPERO as CRD42021257984.

## Introduction

Current dietary guidelines generally recommend that total fat intake should not exceed 35% of total daily energy intake [1]. However, fat intake associated with a modern Western diet can exceed this recommendation [2], contributing to the rising rates of obesity and type 2 diabetes [3]. The link between the consumption of a high-fat diet (HFD) and the development of metabolic disease is multifactorial, but ectopic storage of excess lipids within skeletal muscle appears to be an important etiologic factor [4].

In resting human skeletal muscle, plasma-derived nonesterified fatty acids (NEFA) enter the intramyocellular lipid (IMCL) pool and are first incorporated into intramuscular triacylglycerols (IMTGs) within lipid droplets before the IMTG-derived fatty acids are released for oxidation [5]. Following HFD, IMTG synthesis can exceed IMTG lipolysis, resulting in the expansion of the IMCL pool [6,7]. However, not all studies observe IMCL accumulation following HFD [8,9].

This discrepancy may be because of sampling errors associated with small sample sizes in individual studies and/or the methodologic variability among studies, such as the following: *1*) the difference in HFD total fat content, *2*) HFD duration, and/or *3*) the measurement techniques used to quantify IMCL content. Transmission electron microscopy [10] and immunofluorescence microscopy can determine fiber-type-specific IMCL content in muscle biopsies [11]. Biochemical estimates of IMCL from mixed muscle biopsy samples do not reveal fiber-type-specific IMCL content and are potentially confounded by extramyocellular lipid [12], contributing to a large variability in the measurement of IMCL content across serial muscle biopsies [13]. Finally, the noninvasive H^1^ magnetic resonance spectroscopy (^1^H-MRS) technique also cannot determine fiber type-specific IMCL content, but it can distinguish between intra- and extramyocellular lipids [13]. Pooling the results of individual studies is needed to resolve discrepancies between studies and to explore potential sources of heterogeneity [14].

IMCL content is negatively associated with insulin sensitivity in healthy individuals [[15], [16], [17]], and following HFD, IMCL accumulation and a reduction in insulin sensitivity have also been reported [18]. Together, these findings may suggest a link between IMCL and insulin sensitivity. However, in endurance-trained individuals, high IMCL content coexists with high-insulin sensitivity [4], casting doubt on the causal relationships between IMCL and insulin sensitivity. It is possible that the storage capacity or turnover of the IMCL pool in response to HFD is a means to reduce the effects of excess lipids on insulin sensitivity [7].

Therefore, the main purpose of this study was to synthesize evidence through a systematic review and meta-analysis of the effect of an HFD on IMCL content in healthy individuals. A secondary aim was to explore the potential modifying effects of HFD total fat content, HFD duration, IMCL measurement technique, and physical activity status on IMCL content. We also aimed to delineate the relationship between HFD-associated changes in IMCL content and insulin sensitivity.

## Methods

This systematic review and meta-analysis was completed in accordance with PRISMA guidelines [19] and was prospectively registered with the PROSPERO database (reference: CRD42021257984).

### Search strategy

A systematic search was conducted by 2 independent reviewers (JA and KJ) on 4 electronic databases: PubMed, Cochrane CENTRAL Register of Controlled Trials, as well as CINAHL and SPORTDiscus via EBSCOhost. The final search was conducted on 1 July 2023 using a combination of synonyms and relevant medical subject headings (MeSH) terms for HFD and IMCL, or triglyceride. Publication date and language restrictions were not applied. The specific keywords and full search strategy for each database can be found in Supplemental Table 1. We also manually screened the reference lists and forwarded citations of included studies, as well as relevant review articles, to identify potentially eligible studies, were also conducted.

### Inclusion criteria

Studies were required to meet the following inclusion criteria: *1*) they were original research studies involving healthy human participants aged 18–64 y. Older adults were excluded because they are more likely than their younger counterparts to be taking multiple prescription medications [20]; *2*) the intervention should involve ≥3 d of high-calorie HFD or normocaloric HFD; *3*) IMCL content was measured after the intervention; *4*) full text was available in English; and *5*) the study was a randomized controlled trial (RCT), within-subject crossover trial, or single-arm pretest-posttest trial. Studies were required to describe the composition of the dietary intervention in adequate detail, where total fat intake was >35% of the total daily energy intake. Studies were excluded if they met the following criteria: *1*) participants were recruited on the basis of any medical condition; *2*) the HFD intervention was delivered in conjunction with exercise or immediately after exercise; *3*) the HFD intervention followed a period of low-fat dietary intake; and *4*) the article has been retracted.

### Study selection

Eligible studies were collected into a single list in Microsoft Excel (Microsoft Corporation). Two reviewers (JA and KJ) independently removed duplicates and reviewed the titles and abstracts to assess eligibility for inclusion. Reviewers were not blinded to the articles, as studies have shown that the summary outcome was not affected by blinding during study selection and data extraction [21]. Articles were initially excluded based on the content of the titles and abstracts, followed by a full-text review by 2 independent reviewers (JA and KJ). Conflicts were resolved through discussion or by a third reviewer (AH) if required.

### Data extraction

Data were extracted independently by 2 researchers (JA and KJ) onto a project-specific data extraction form (Microsoft Excel). The data items extracted were as follows: *1*) study characteristics (design, location, and diet duration), *2*) participant characteristics (sample size, sex, age, body mass, BMI (in kg·m^–2^), participant health status, and participant training status), *3*) details of the intervention (composition of the diet, control, duration, and number of participants per trial arm) and control arm (if applicable), and *4*) details of the IMCL measurement techniques. Preintervention, postintervention, and change score data for the primary outcome (IMCL content) were also extracted [mean and (SD)]. For studies that reported fiber type-specific responses in IMCL content, data for type I and type II muscle fiber IMCL responses were extracted. For studies that reported the IMCL content within multiple muscles, data for IMCL responses in each muscle were extracted. Additionally, pre and postintervention data were extracted for the following secondary outcome measures: insulin sensitivity, circulating concentrations of fasting glucose, insulin, triacylglycerol (TAG), and NEFA. If SDs were not reported, we collected other relevant data that can be converted to an SD, such as SEs, 95% confidence intervals (CIs), or *P* values. When values were presented in figure form only, the figure was digitized using graph digitizer software [22], and the means and SD/SEM were measured manually at the pixel level to the scale provided. However, for missing and unreported data or any further details, corresponding authors of studies were contacted via email on 2 occasions within a 1-mo period.

### Assessment of risk of bias in included studies

Two authors (JA and AG) independently assessed the risk of bias in included studies in accordance with the recommendations of the Cochrane Handbook for Systematic Reviews of Interventions [23]. Cochrane Risk of Bias tool (RoB 2) was used to evaluate randomized trials [24], and the risk of bias in non-randomised studies - of interventions (ROBINS-I) tool was used to evaluate nonrandomized trials [25]. The risk of bias in studies that employed a crossover design was evaluated using RoB 2 for consistency. Disagreements between reviewers were resolved by consensus with a third reviewer (AH).

The risk of bias because of missing results in a meta-analysis was explored with Egger’s test of the intercept and by visually inspecting a funnel plot of the treatment effects plotted against their corresponding sampling variance.

### Statistical analysis

If 2 or more studies reported the same outcome, a meta-analysis of standardized mean differences (SMDs) was performed [26]. For studies that used independent groups, SMDs were calculated as the mean difference divided by the pooled baseline SD, where the mean difference is calculated as the mean pre–post change in the intervention group minus the mean pre–post change in the control group [27]. For studies that used matched groups (crossover trials and single-arm pre–post trials), SMDs were standardized using the change score SD. If the change score SD was not reported, it was estimated using the standard formula [28] and assuming a within-groups correlation of 0.7 [[29], [30], [31]]. From a statistical perspective, the SMD has the same meaning regardless of study design, and thus, we followed guidelines for including different study designs in the same meta-analysis [32]. Hedges’ g correction was applied to SMDs. Interpretation of effect size was as follows: <0.20 as trivial, 0.20–0.49 as small, 0.50–0.79 as moderate, and >0.80 as large [33].

Meta-analyses were performed with a random effects model using the restricted maximum likelihood method to estimate between-study variance. CIs and test statistics were calculated via a t-distribution using the Hartung-Knapp-Sidik-Jonkman approach [34]. Studies were weighted according to the inverse of the sampling variance. If a meta-analysis included >1 outcome measure from the same study, effect estimates were nested within studies using a multi-level structure [35]. Statistical heterogeneity between studies was evaluated with the χ^2^ test and the *I*^2^ statistic. Thresholds for the interpretation of *I*^2^ were in line with Cochrane recommendations: 0–40% (“might not be important”), 30%–60% (“moderate heterogeneity”), 50%–90% (“substantial heterogeneity”), and 75%–100% (“considerable heterogeneity”) [14]. The importance of the observed *I*^2^ value was interpreted alongside its 95% CI and the *P* value from the χ^2^ test [14]. Graphic Display of Heterogeneity plots were used to investigate between-study heterogeneity and identify potentially influential studies. Statistical analyses were conducted using package metafor in R version 4.2 (R Foundation for Statistical Computing). If a meta-analysis model includes ≥10 effect estimates [14], sources of heterogeneity with meta-regressions were explored. Covariates were included: IMCL measurement technique, participant physical activity status, study design, HFD duration, HFD total fat content, composition of total fat intake, and participant body mass.

## Results

### Literature search

**FIGURE 1:**
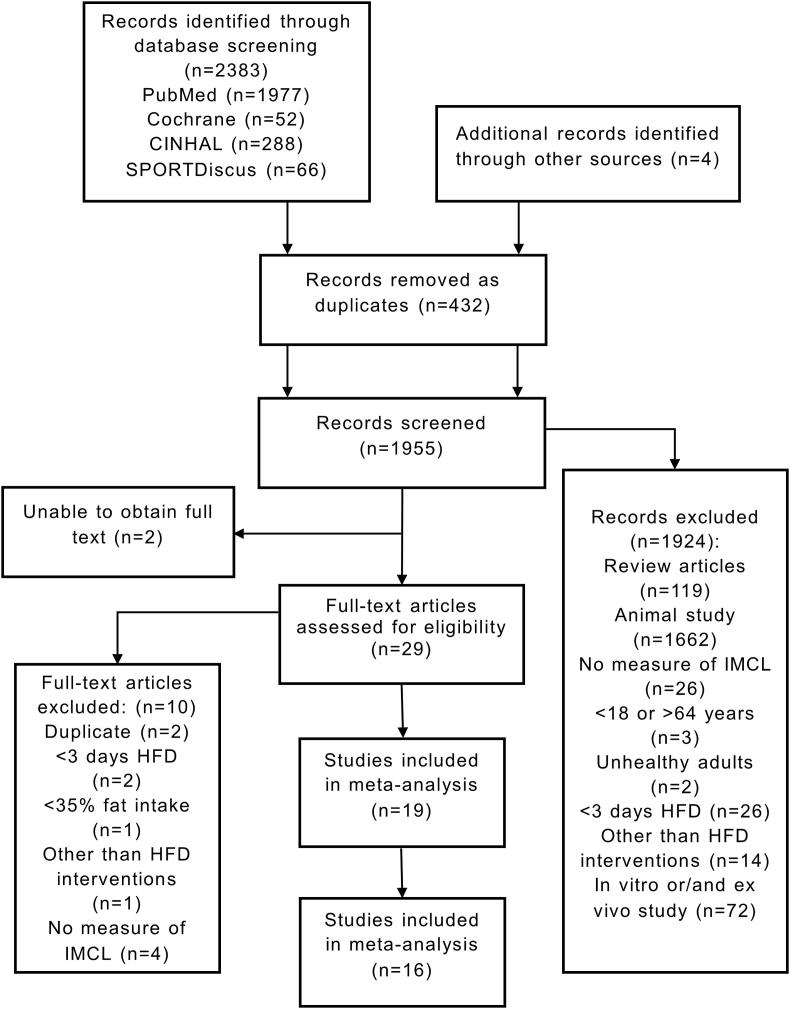
Flow chart of study selection. HFD, high-fat diet; IMCL, intramyocellular lipid.

A total of 2383 articles were identified, which was reduced to 1955 after removing duplicates. Following the screening of titles and abstracts, 1926 studies were excluded because they did not meet the eligibility criteria, and we were unable to access the full text of 2 studies. Following the appraisal of full texts, a total of 19 studies were suitable for inclusion in the systematic review. Three studies [[36], [37], [38]] could not be synthesized by meta-analysis because of insufficient data; thus, 16 studies were included in the meta-analysis (Figure 1). Six studies were controlled trials, and 10 were single-arm trials. Of the 6 controlled trials, 3 were parallel-groups, and 3 were crossover-designed trials.

### Study characteristics

A total of 303 participants were included in the meta-analysis with a mean age of 28 y (range 21–44 y). Individual study characteristics and their main findings are summarized in Table 1. The mean BMI at baseline was 24.5 (range 20.6–30.1); however, only 1 study included participants with a BMI >25 [9]. Regarding the techniques used to measure IMCL, 6 studies used ^1^H-MRS [6,18,[39], [40], [41], [42]], 5 studies used biochemical extraction [[43], [44], [45], [46], [47]], and 5 used microscopy, of which 1 study used transmission electron microscopy [48] and 4 studies used immunofluorescence microscopy [[7], [8], [9],^49^]. One study used both ^1^H-MRS and microscopy [8].

**TABLE 1:** Summary of baseline data and effects of the high-fat diet on intramyocellular lipid content in the intervention group

Author	*N* (sex)	Participant characteristics (age; y)	HFD total fat content	Intervention diet	Comparator diet total fat content	Study design	Duration (day)	Measurement techniques	Major findings (IMCL content)
Adochioet al. [36]	M = 11 F = 10	Healthy lean (28)	50%, 156 g	Hypercaloric HFD (+40%)	30%, 67 g	Crossover (3 phases)	5	^1^H-MRS	↑
Cornford et al. [45]	M = 7 F = 2	Healthy, non-obese, physically inactive (24)	35%, 155 g	Hypercaloric HFD	NC	Single-arm trial	14	Biochemical extraction	↔
Covington et al. [8]	M = 29	Healthy, physically active (27)	44%	Hypercaloric HFD (140%)	NC	Single-arm trial	56	Microscopy, Oil red O stain	↔
Gemmink et al. [46]	M = 12	Healthy, lean Caucasian, and South Asian (22)	94%	Hypercaloric HFD	NC	Single-arm trial	5	Biochemical extraction	↑
Hoppeler et al. [38]	M = 7	Well-trained runners (37)	41%,	Hypercaloric HFD	18%	Crossover (2 phases)	28–31	Transmission electron microscopy	↑
Johannsen et al. [18]	M = 29	Healthy (27)	44%, 207 g	Hypercaloric HFD (+40%)	NC	Single-arm trial	56	^1^H-MRS	↔
Kadowaki et al. [42]	M = 21	Healthy, non-obese (25)	48%, 320 g	Hypercaloric HFD (+45%)	NC	Single-arm trial	6	^1^H-MRS	↑
Kakehi et al. [41]	M = 50	Healthy, physically active (23)	60%	Normocaloric HFD	NC	Single-arm trial	3	^1^H-MRS	↑
Kien et al. [37]	M = 12 F = 12	Healthy, non-obese (29)	42%	Normocaloric high-palmitic acid diet and high-oleic acid diet	34%	Randomized parallel-arm trial (2 arms)	7	Biochemical extraction	↔
Kiens et al. [43]	M = 10	Healthy, nonobese, physically active (36)	54%	Normocaloric HFD	43%	Randomized parallel-arm trial (3 arms)	28	Biochemical extraction	↑
Lundsgaard et al. [47]	M = 9	Healthy, moderately trained (23)	78%, 496 g	Hypercaloric HFD, hypercaloric LFD, and Normocaloric NFD	24%, 87 g	Randomized crossover (3 phases)	3	Biochemical extraction	↑
Schrauwen-Hinderling et al. [39]	M = 7	Healthy (25)	60%, 218 g	Normocaloric NFD followed by Normocaloric HFD	NC	Single-arm trial	7	^1^H-MRS	↑
Skovbro et al. [44]	M = 21 (I = 10, CON = 11)	Healthy, untrained (24)	58%, 177 g	Normocaloric HFD and Normocaloric NFD	30%, 76 g	Randomized parallel-arm trial (2 arms)	17.5	Biochemical extraction	↔
St-Onge et al. [40]	M and F = 24	Healthy overweight (44)	37%	Normocaloric HFD, Normocaloric HPUFA, and Normocaloric NFD	31%	Randomized crossover (3 phases)	25	^1^H-MRS	↑
Suzuki et al. [6]	M = 42	Healthy, non-obese, and physically active (23)	60%	Normocaloric HFD	NC	Single-arm trial	3	^1^H-MRS	↑
Tsintzas et al. [9]	M = 9	Healthy overweight/obese, physically inactive (44)	49%,	Hypercaloric HFD (+25%)	NC	Single-arm trial	14	Microscopy, LD540 stain	↔
Van Proeyen et al. [49]	M = 7	Healthy, physically active (21)	50%, 123 g	Hypercaloric HFD (+30%)	34%, 113 g	Randomized parallel-arm trial (3 arms)	42	Microscopy, Oil red O stain	↑
Vogt et al. [48]	M = 11	Healthy, duathletes (32)	53%, 192 g	Normocaloric HFD (+12%) and Normocaloric LFD	17%, 53 g	Randomized crossover (2 phases)	35	Transmission electron microscopy	↑
Whytock et al. [7]	M = 11 F = 2	Healthy, lean, physically active (23)	64%, 325 g	Hypercaloric HFD (+47%)	NC	Single-arm trial	7	Microscopy, BODIPY stain	↑

^1^H-MRS, H^1^ magnetic resonance spectroscopy; BODIPY, boron dipyrromethene; CON, control; F, female; HFD, high-fat diet; HPUFA, high-polyunsaturated fat; I, intervention; IMCL, intramyocellular lipids; LD540, Lipophilic fluorescent dye; LDL, low density lipoprotein; LFD, low-fat diet; M, male; NC, no comparator; NFD, normal-fat diet; BODIPY; LD540.

↑: increased; ↔: unchanged. Because of missing data in some studies, values were not included.

### Diet composition

In all studies, the intervention group consumed the HFD, and the comparator groups consumed either a low-fat diet or a habitual normal-fat diet. Participants in 9 studies consumed hypercaloric HFD, whereas in 7 studies normocaloric HFD. Intervention groups consumed HFD with a proportion of fat between 35% and 78% of total daily energy expenditure. Across all studies, the mean total fat intake in HFD was 243 g/d (range 123–496 g/d). The HFDs provided across studies were either high in saturated fat (>10% of daily calories) [9,46], high in polyunsaturated fat (≤10% of daily calories) [40], and high in unsaturated fat (≤30% of daily calories) [47]. Intervention duration ranged from 3 to 56 d.

### IMCL content

**FIGURE 2:**
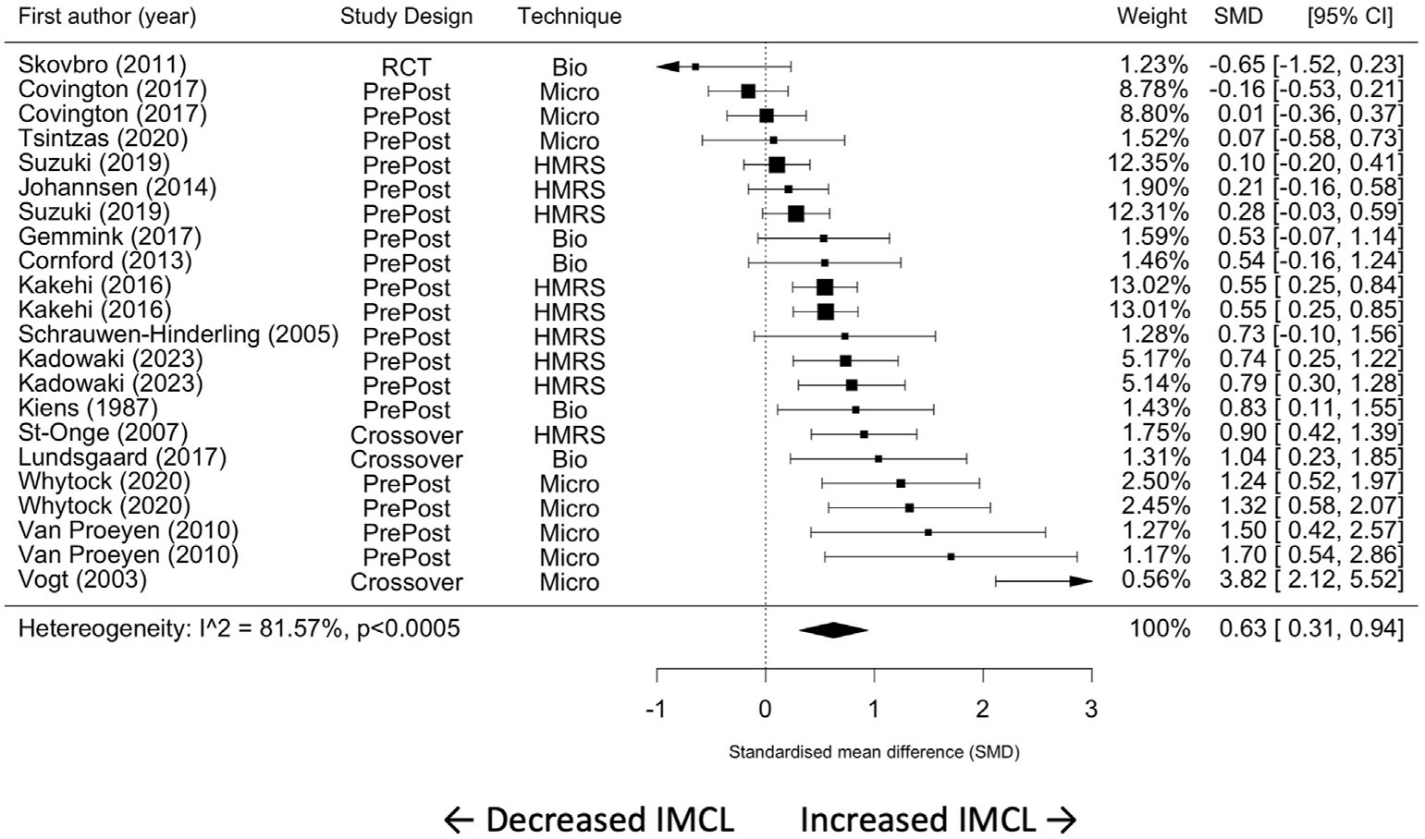
Forest plot of the effect of an HFD on IMCL content in healthy individuals. CI, confidence interval; HFD, high-fat diet; IMCL, intramyocellular lipid; SMD, standardized mean difference; ^1^HMRS, H^1^ magnetic resonance spectroscopy.

**FIGURE 3:**
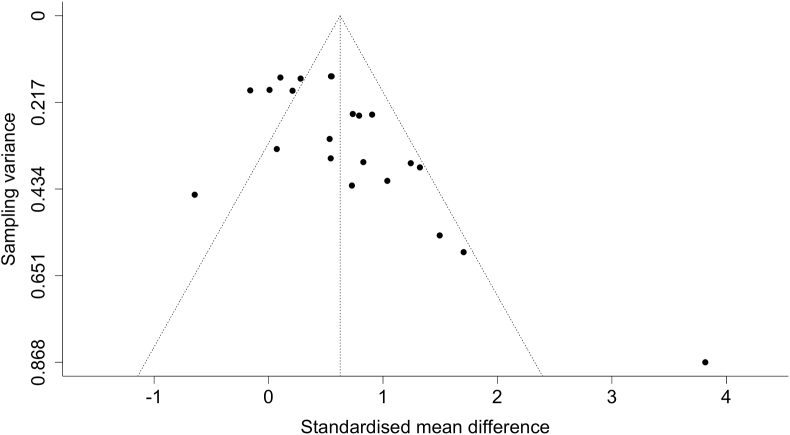
Funnel plot for the included studies in the meta-analysis.

A meta-analysis comprising 22 effect estimates from 16 studies showed that IMCL content was significantly increased following HFD (SMD = 0.63; 95% CI: 0.31, 0.94, *P* < 0.001; Figure 2). This effect size corresponds to a percentage increase of 31.4% ± 29.2%. The magnitude of heterogeneity in IMCL responses to HFD was substantial between these studies (*I*^2^ = 81.57%; *P* < 0.001). Inspection of the funnel plot showed asymmetry in the distribution of the studies included in the meta-analysis, and Egger’s regression test indicated evidence of small study effects (β = 4.07; 95% CI: 1.91, 6.22; *P* < 0.001; Figure 3), both of which may indicate a potential publication bias or small-study effect. Following HFD, there was an increase in IMCL content in studies using the microscopy technique (37.9% ± 37%), ^1^H-MRS (29.1% ± 16.5%), or Biochemical extraction (23.1% ± 36.6%). However, meta-regression revealed that the determination of IMCL responses to HFD was not affected by the IMCL measurement technique (*P* > 0.05) (Table 2).

**TABLE 2:** Summary of meta-regression results

Outcome	Covariate	Coefficient (95% CI)	*P* value	*I*^2^ (χ^2^*P* value)
IMCL content	Measurement technique ^1^H-MRS and Biochemical extraction^1^ Microscopy (*n* = 5)	0.41 (–0.33, 1.15)	0.26	83.92% (<0.0001)
	Microscopy and Biochemical extraction^1^ ^1^H-MRS technique (*n* = 6)	–0.16 (–0.83, 0.51)	0.61	83.76% (<0.0001)
	^1^H-MRS and microscopy^1^ Biochemical extraction technique (*n* = 5)	–0.21 (–0.95, 0.53)	0.55	83.81% (<0.0001)
	Study design (*n* = 16) RCT and crossover trials^1^ Pre-post trials	–0.33 (–1.16, 0.49)	0.41	83.12% (<0.0001)
	HFD duration (*n* = 16)	–0.001 (–0.01, 0.016)	0.84	83.5% (<0.0001)
	Fat content (*n* = 10)	0.001 (–0.005, 0.006)	0.83	84.68% (0.0001)
	Body mass (*n* = 13)	–0.07 (–0.22, 0.07)	0.29	84.15% (0.0001)
	Physical activity status (*n* = 12) Inactive^1^ Active	0.42 (–0.23, 1.09)	0.19	83.44% (<0.0001)
	Energy intake (*n* = 16) Normocaloric diet^1^ Hypercaloric diet	–0.02 (–0.70, 0.65)	0.94	83.82% (<0.0001)
Hyperinsulinemic-euglycemic clamp	IMCL (*n* = 7)	–0.38 (–1.09, 0.34)	0.23	0% (0.71)
HOMA-IR	IMCL (*n* = 4)	0.43 (–1.03, 1.88)	0.33	0% (0.96)

HFD, high-fat diet; HOMA-IR, homeostatic model assessment for insulin resistance; IMCL, intramyocellular lipids; *n*, number of effect estimates; RCT, randomised controlled trial; ^1^H-MRS, H^1^ magnetic resonance spectroscopy; 95% CI, 95% confidence interval; χ^2^,chi-squared test.

1 Reference category in the model.

### Circulating triglyceride, and NEFA

**FIGURE 4:**
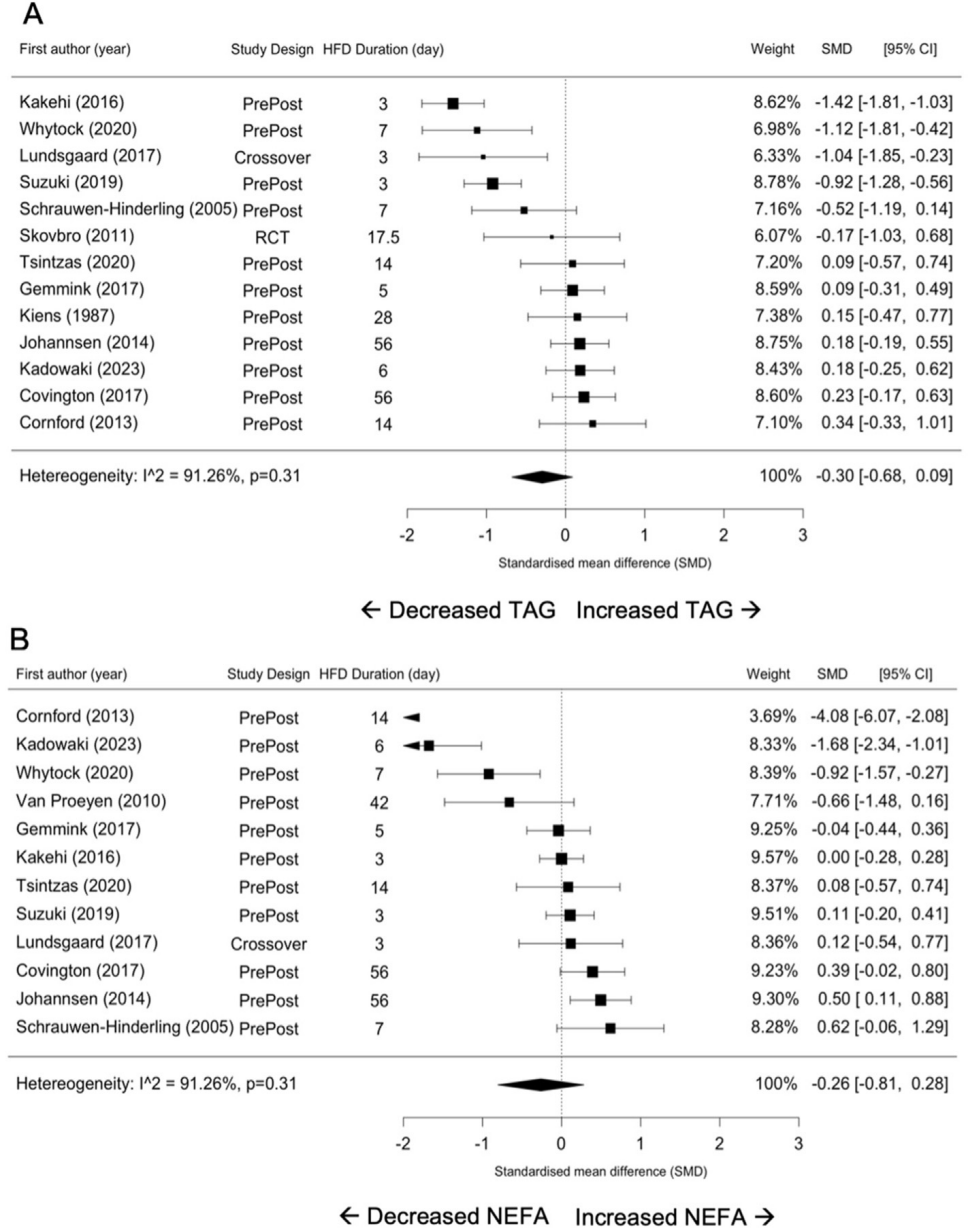
Forest plots of the effects of an HFD on circulating TAG (A) and NEFA (B). CI, confidence interval; HFD, high-fat diet; NEFA, nonesterified fatty acid; SMD, standardized mean difference; TAG, triacylglycerol.

Fasting circulating concentration of TAG (SMD = –0.30; 95% CI: –0.68, 0.09; *P* = 0.12 Figure 4A) nor NEFA (SMD = –0.26; 95% CI: –0.81, 0.28; *P* = 0.31; Figure 4B) changed in response to HFD. There was considerable heterogeneity between studies for both circulating TAG and NEFA concentrations (*I*^2^ = 83.24%; *P* < 0.001 and *I*^2^ = 91.26%; *P* < 0.001, respectively). The magnitude of heterogeneity was not affected by the study characteristics as covariates in the meta-analysis. However, physical activity status contributed to changes in circulating TAG concentration (*P* = 0.03).

### Insulin sensitivity

**FIGURE 5:**
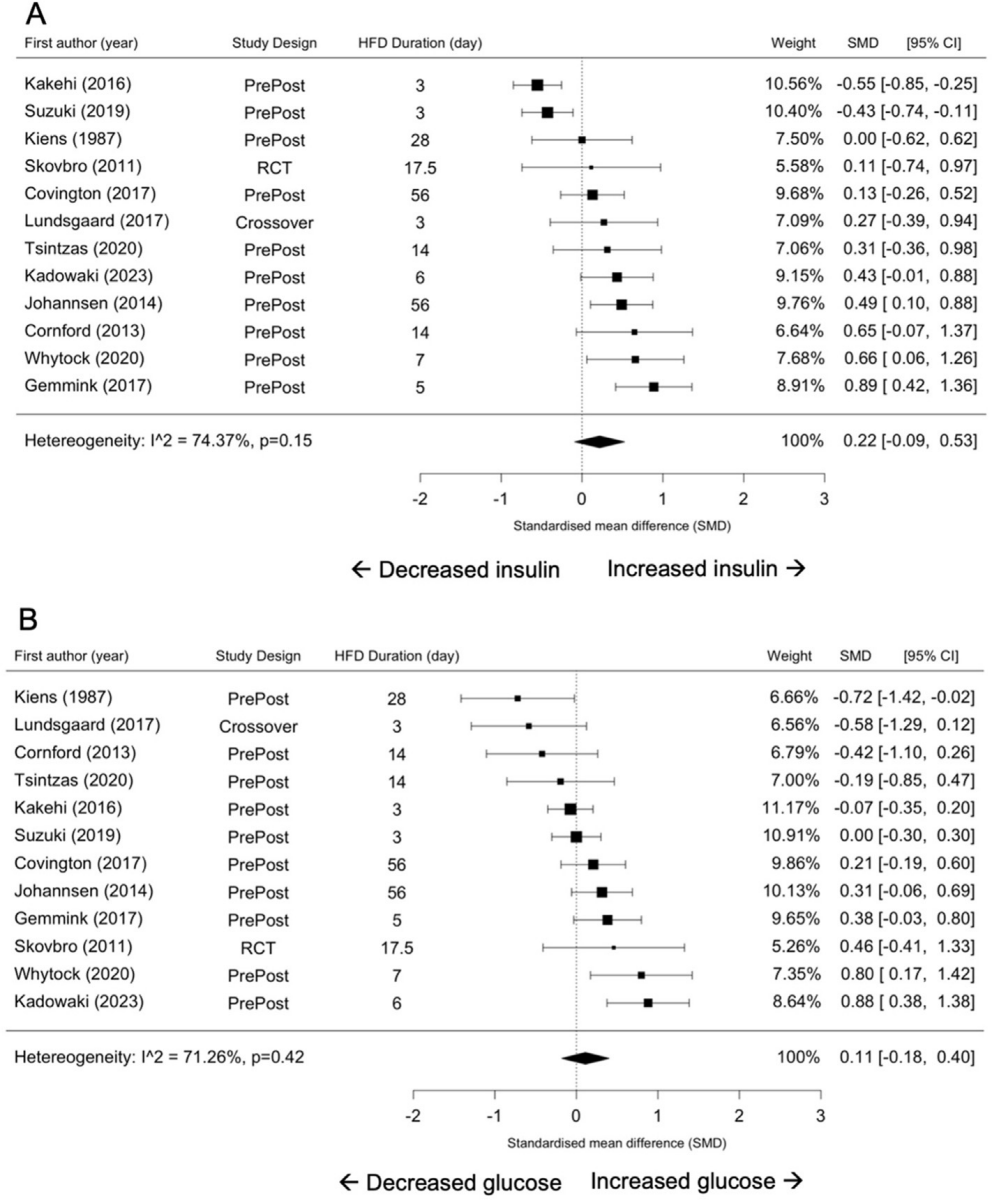
Forest plots of the effects of an HFD on circulating insulin (A) and glucose (B). CI, confidence interval; HFD, high-fat diet; SMD, standardized mean difference.

HFD did not affect fasting concentrations of circulating insulin (SMD = 0.22; 95% CI: –0.09, 0.53; *P* = 0.15; Figure 5A) and glucose (SMD = 0.11; 95% CI: –0.18, 0.40; *P* = 0.42; Figure 5B). There was a substantial and considerable degree of heterogeneity across studies that reported fasting circulating insulin (*I*^2^ = 74.37%; *P* < 0.001) and glucose concentration (*I*^2^ = 71.26%; *P* < 0.001), respectively.

**FIGURE 6:**
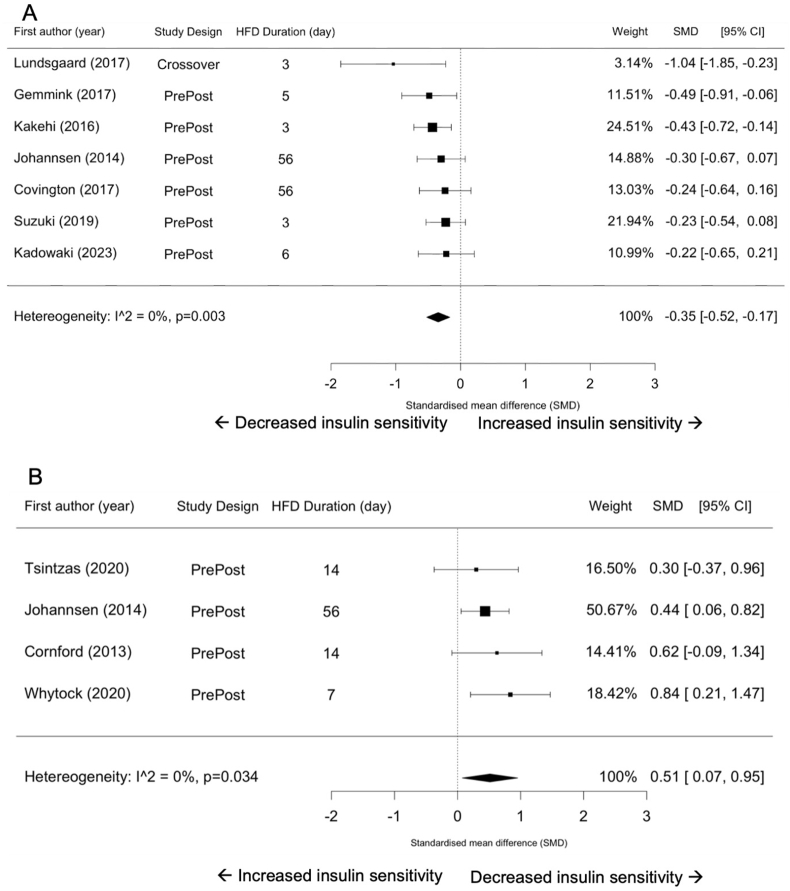
Forest plots of the effects of an HFD on hyperinsulinemic-euglycemic clamp (A) and HOMA-IR (B). CI, confidence interval; HFD, high-fat diet; SMD, standardized mean difference.

Seven studies used the hyperinsulinemic-euglycemic clamp technique to measure insulin sensitivity [6,8,18,41,42,46,47], and 4 used the HOMA-IR [7,9,18,45]. Insulin sensitivity decreased in response to a HFD, as determined by the hyperinsulinemic-euglycemic clamp (SMD = –0.35; 95% CI: –0.52, –0.17; *P* = 0.003; Figure 6A), and HOMA-IR (SMD = 0.51; 95% CI: 0.07, 0.95; *P* = 0.03; Figure 6B). The degree of heterogeneity might not be important between studies for hyperinsulinemic-euglycemic clamp (*I*^*2*^ = 0.00%; *P* = 0.57) and HOMA-IR (*I*^2^ = 0.00%; *P* = 0.64).

### Meta-regressions

Meta-regressions for the effect of IMCL measurement techniques, study design, HFD duration, HFD total fat content, change in body mass, physical activity status, calorie intake, and change in IMCL content on outcomes are presented in Table 2 and Supplemental Table 2. Physical inactivity and higher energy intake had a modifying effect on circulating insulin (*P* = 0.02; 0.01, respectively). Furthermore, higher energy intake was associated with a greater reduction in insulin sensitivity measured by HOMA-IR (*P* = 0.03). Additionally, physical activity was associated with the change in circulating TAG concentration (*P* = 0.03). All other meta-regressions showed no significant modifying effects on outcomes.

### Risk of bias

**FIGURE 7:**
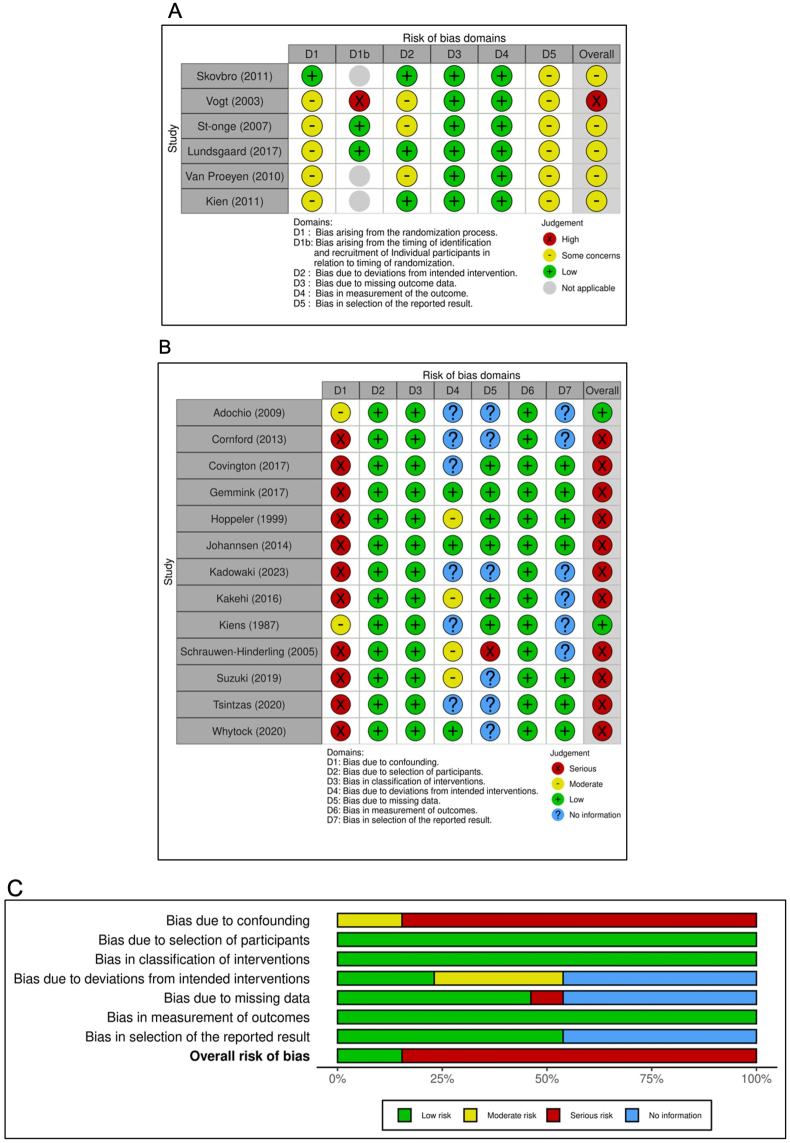
Assessment of risk of bias for randomized studies using the Cochrane RoB 2 tool (Traffic-light plot) (A), nonrandomized studies using the Cochrane ROBINS-I tool (Traffic-light plot) (B), and nonrandomized studies using the Cochrane ROBINS-I tool (Summary plot) (C). RoB, risk of bias.

Six randomized studies were assessed by the RoB 2 tool, and 13 nonrandomized studies were assessed by the ROBINS-I tool. Of the 6 randomized studies, 5 studies were judged to raise some concerns about the overall risk of bias [37,40,44,47,49], and 1 was considered to have a high overall risk of bias because of not including a washout period [48]. Five randomized studies did not provide information on sources of recruitment [37,40,[47], [48], [49]]. Of the 13 nonrandomized studies, 2 studies were judged to have an overall low risk of bias [36,43], and 11 studies were considered to have a high overall risk of bias [[6], [7], [8], [9],^18^,^38^,^39^,^41^,^42^,^45^,^46^] because of not controlling for confounding variables in the analysis. Detailed judgments for each domain in each included study are presented in Figure 7A, B, and C.

## Discussion

The purpose of this systematic review and meta-analysis was to provide novel evidence on the effect of ≥3-d HFD on IMCL content and the association between changes in IMCL content and insulin sensitivity in healthy individuals. Evidence suggests that *1*) IMCL is accumulated in healthy individuals following an HFD with a duration of between 3 and 56 d; *2*) IMCL accumulation was not associated with HFD total fat content, duration, physical activity status, or measurement technique; *3*) insulin sensitivity is reduced following HFD, but this was not related to IMCL accumulation.

The increase in IMCL content showed a moderate effect size in response to HFD. This agrees with the findings of a 7-study meta-analysis, where IMCL content increased following HFD [50]. This confirms that IMCL accumulation is a means by which excess dietary lipids can be accommodated [51]. Our findings are potentially explained by acute adjustments within the skeletal muscle to an HFD, which include a rapid upregulation of IMCL synthesis rates in response to elevated fatty acid availability [52] and chronic adaptations that favor enhanced fatty acid transport [53,54] and IMCL storage [7].

A novel finding of the present meta-analysis was that HFD total fat content was not associated with the increase in IMCL in response to HFD. This suggests that consuming a diet >35% of total energy intake elicits an increase in IMCL content, but greater fat intake may not further increase IMCL accumulation. Meta-regression analysis also revealed that the duration of HFD was not associated with the increase in IMCL content. This means that a longer duration of HFD, beyond a minimum of 3 d, does not further increase IMCL accumulation. Together, these novel findings suggest that after as little as 3-d of a modest over-supply of lipids, IMCL stores may reach capacity in healthy individuals and that there must be an alternative fate for the continued over-supply of lipids other than storage as IMCL. However, meta-regression analysis may lack sensitivity because of the small number of studies that examined the effect of total fat content and duration on IMCL responses to HFD. In addition, bias within individual studies, visual inspection of the funnel plot, and Egger’s test of the intercept were suggestive of small-study effects, which may correspond to issues with publication and/or reporting bias, among other issues. To validate these findings, more experimental research is required to examine the time course of IMCL responses to HFD with varying fat content and/or duration.

The present study found no change in fasting circulating concentrations of TAG and NEFA following HFD. This indicates that an over-supply of lipids through high-fat feeding is countered by increased clearance of circulating TAG and NEFA. In support, an unchanged serum TAG concentration following a 4-wk HFD in healthy individuals coincided with increased skeletal muscle lipoprotein lipase activity and IMCL content [43]. The maintenance of circulating TAG and NEFA at baseline concentrations, despite continued HFD, occurred without further expansion of the IMCL pool. This may be explained by an increased rate of IMCL turnover [5,55,56], fat oxidation [57,58], and/or lipid storage within other tissues, such as hepatic [59], and subcutaneous, visceral and/or intermuscular adipose tissues [60].

To our knowledge, the present meta-analysis is the first to assess the modifying effect of study characteristics on IMCL responses to HFD. Endurance-trained individuals with a high IMCL content have a higher resting IMCL synthesis rate [61], IMCL turnover rate[62], and a greater oxidation rate of IMCL-derived fatty acids [[63], [64], [65]] compared with others. Therefore, we hypothesized that physically active individuals would have a greater capacity for lipid uptake, IMCL turnover, and fatty acid oxidation when challenged with HFD. This would enable lipid clearance despite a limited capacity for IMCL storage. Contrary to this hypothesis, meta-regression analysis showed no modifying effect of physical activity level on the IMCL response to HFD. However, the inclusion criteria employed by the present study may have resulted in a rather homogeneous sample in relation to physical activity. Further, physical activity was inconsistently measured and reported in some of the studies included, evidenced by the considerable between-study heterogeneity. As such, further research is required to elucidate the effect of physical activity on the ability of skeletal muscle to tolerate and accommodate HFD.

Biochemical extraction, ^1^H-MRS, and microscopy were the 3 measurement techniques used by studies to quantify IMCL content, and this may have contributed to the substantial heterogeneity we observed between studies in IMCL responses to HFD. For the first time using meta-regression analysis, we demonstrate no effect of the IMCL measurement technique on the assessment of change in IMCL content following HFD. This suggests that each of the 3 IMCL measurement techniques can be used to determine IMCL responses to HFD. This is surprising, as the IMCL measurement technique can affect the measurement of IMCL content at rest [66] and in the change in IMCL content following acute exercise [67]. Our finding may be explained by the significant increase in IMCL content following HFD being of a sufficient magnitude to minimize the effect of measurement technique on assessing the change in IMCL content. However, caution is needed because of the relatively low number of included studies in this meta-regression.

We found a reduction in insulin sensitivity following HFD. However, meta-regression analysis revealed that the reduction in insulin sensitivity was not associated with the increase in IMCL content following HFD. This is unsurprising because it is well-accepted that a high-IMCL storage per se does not necessarily lead to insulin resistance [16]. A lower turnover of IMCL in physically inactive individuals [61] and those with excess adiposity [55] compared with more physically active individuals can result in the accumulation of fatty acid metabolites within skeletal muscle, such as diacylglycerol [68] and ceramide [69] and it is these which appear to exert the lipotoxic effect on skeletal muscle insulin signaling [70,71]. However, meta-regression analysis revealed that insulin sensitivity following HFD was not modified by physical activity status. In addition, of the included studies, evidence is mixed for an effect of HFD on diacylglycerol [7,9,47,49] and ceramide content [[7], [8], [9],^47^,^49^] in healthy individuals, suggesting further research is required. It is likely that excess lipid was taken up by subcutaneous, visceral, and/or intermuscular adipose tissues [60]. HFD-induced adipocyte hypertrophy has been shown to elicit tissue-specific [72,73] and whole-body [74] insulin resistance. In addition, intermuscular adipose tissue is negatively associated with insulin sensitivity [75] and may have contributed to the reduction in insulin sensitivity in the present study [76]. To what extent HFD affects intermuscular adipose tissue metabolism and insulin sensitivity in healthy individuals warrants further investigation.

This meta-analysis provides the most comprehensive and contemporary review to date, comprising 22 effect estimates from 16 studies assessing IMCL responses to HFD. It also included meta-regression analysis to investigate the potential sources of heterogeneity. Although this meta-analysis yields new, important evidence, some limitations to the current body of literature warrant consideration. Approximately 70% of the studies included were single-arm, pre-post studies, for which the risk of bias is inherently greater than the stronger study design of an RCT. Only ∼6% of studies recruited female participants, and they did not report sex-specific effects of HFD on IMCL content. As it has been shown that females have higher IMCL accumulation in response to lipid infusion [77,78], it is possible that skeletal muscle lipid storage in response to HFD may differ between males and females. Because females are under-represented in this research, more studies are required to determine the sex-specific effects of an HFD on IMCL content. In RCT and crossover designs studies, the difference in fat content between the intervention and comparator diets was small. This could mean that the effect size of HFD on IMCL content was underestimated compared with normal-fat and low-fat diets. The effect of monounsaturated, polyunsaturated, and saturated fats on IMCL responses to HFD was not explored in the present meta-analysis. This is because only 1 study in the current meta-analysis investigated the effect of a high-polyunsaturated fat diet on IMCL content [40]. It is possible that IMCL content responds differently to a diet high in polyunsaturated, monounsaturated, or saturated fat [37,40]. The present study did not investigate the effect of ethnicity on the IMCL response to HFD in different ethnic groups because of the lack of racial and ethnic diversity in the included studies. Future experimental studies should investigate the effect of HFD on IMCL content in different ethnic groups [79,80]. The present study explored IMCL responses to HFD in healthy adults aged <65 y, so our findings cannot be generalized to older adults and other individuals who possess insulin resistance, type 2 diabetes, and other chronic diseases.

In conclusion, this meta-analysis confirms that IMCL content is increased following an HFD in healthy individuals. Although an HFD does increase IMCL content, our data would suggest that the duration – beyond 3 d – and the fat content – beyond an intake exceeding 35% of daily energy from fat – do not influence IMCL accumulation. No effect of measurement techniques on the change in IMCL content was shown in response to HFD. Furthermore, a significant reduction in insulin sensitivity in response to HFD was observed, yet this reduction was not associated with the increase in IMCL content. Future well-designed trials are needed to improve the overall quality of evidence and the precision of the effect estimates.

## Author contributions

The authors’ responsibilities were as follows – JA, OW, AS-K, AH, JM: were responsible for the conceptualization and development of the study; JA, KJ: performed the systematic searches, removed duplicates, screened the abstracts, assessed full texts for eligibility, and extracted outcome data; JA, AG: performed the risk of bias assessment; JA, STO: conducted the statistical analyses; JA, OW, AS-K, AH: interpreted the results; JA, OW: wrote the manuscript. OW: had primary responsibility for the final content, and all authors: read and approved the final manuscript.

### Conflict of interest

The authors report no conflicts of interest.

### Funding

The authors reported no funding received for this study.

### Data availability

Data will be made available upon request.
