# Supplementary material for: The Effect of High-Fat Diet on Intramyocellular Lipid Content in Healthy Adults: A Systematic Review, Meta-Analysis, and Meta-Regression
Source: J Nutr. 2024 Feb 26;154(4):1087–100. doi: 10.1016/j.tjnut.2024.02.026 (PMC11007750; doi:10.1016/j.tjnut.2024.02.026)
Supplement: Multimedia component 1 [file mmc1.docx]

**Supplementary Table 1.** Search terms used in each database.

| **PubMed**  ("diet, high fat"[MeSH Terms] OR "diet, carbohydrate restricted"[MeSH Terms] OR (("diet"[All Fields] OR “intake”[All Fields] OR “consumption”[All Fields] OR “ingestion”[All Fields]) AND (“high fat”[All Fields]))) AND (("intramuscular"[All Fields] OR "Intramyocellular"[All Fields] OR "intracellular"[All Fields]) AND (“lipids”[MeSH] OR "lipid"[All Fields] OR “fat”[All Fields] OR "triglycerides"[MeSH Terms] OR "triglycerides"[All Fields] OR "triglyceride"[All Fields])) |
| --- |
| **SPORTDiscus, and CINAHL via EBSCOhost**  S1: (MH "diet, high fat") OR (MH "diet, carbohydrate restricted")  S2: (“diet” OR “intake” OR “consumption” OR “ingestion”)  S3: “high fat”  S4: (“intramuscular” OR “intramyocellular" OR "intracellular")  S5: (MH “Lipids”) OR (MH “Triglycerides”) OR (MH “Lipid Droplets”)  S6: ("lipid" OR "fat" OR "triglycerides" OR "triglyceride" OR "Lipid Droplets")  S7: (S1 OR (S2 AND S3)) AND S4 AND (S5 OR S6) |
| **Cochrane library**  #1 MeSH descriptor: [Diet, High-Fat] explode all trees  #2 MeSH descriptor: [Diet, Carbohydrate-Restricted] explode all trees  #3 MeSH descriptor: [Lipids] explode all trees  #4 MeSH descriptor: [Triglycerides] explode all trees  #5 ("diet" OR "intake" OR "consumption" OR "ingestion"):ti,ab,kw AND ("high fat"):ti,ab,kw  #6 ("intramuscular" OR "intramyocellular" OR "intracellular"):ti,ab,kw  #7 ("lipid" OR "fat" OR "triglycerides" OR "triglyceride" OR "Lipid Droplets"):ti,ab,kw  #8 #1 OR #2 OR #5  #9 #3 OR #4 OR #7  #10 #6 AND #9  #11 #8 AND #10 |
