# Supplementary material for: The Effect of High-Fat Diet on Intramyocellular Lipid Content in Healthy Adults: A Systematic Review, Meta-Analysis, and Meta-Regression
Source: J Nutr. 2024 Feb 26;154(4):1087–100. doi: 10.1016/j.tjnut.2024.02.026 (PMC11007750; doi:10.1016/j.tjnut.2024.02.026)
Supplement: Multimedia component 2 [file mmc2.docx]

**Supplementary Table 2.** Summary of meta-regression results

| **Outcome** | **Covariate** | **Coefficient (95% CI)** | ***p*-value** | ***I*^2^ (χ^2^ p-value)** |
| --- | --- | --- | --- | --- |
| Circulating NEFA | **Study design (n=12)**  RCT and crossover trials^a^  Pre-post trials | -0.42 (-2.57, 1.71) | 0.66 | 92.56% (<0.0001) |
|  | HFD duration (n=12) | 0.01 (-0.01, 0.03) | 0.44 | 91.9% (<0.0001) |
|  | Fat intake (n=8) | 0.002 (-0.008, 0.012) | 0.65 | 94.55% (<0.0001) |
|  | Body mass (n=10) | 0.09 (-0.18, 0.36) | 0.47 | 93.78% (<0.0001) |
|  | **Physical activity status (n=12)**  Inactive^a^  Active | 0.29 (-0.91, 1.49) | 0.59 | 92.74% (<0.0001) |
|  | **Energy intake (n=12)**  Normocaloric diet^a^  Hyper-caloric diet | -0.67 (-1.92, 0.58) | 0.25 | 91.53% (<0.0001) |
|  | IMCL (n=12) | -0.66 (-1.68, 0.36) | 0.18 | 89.09% (<0.0001) |
| Circulating TAG | **Study design (n=13)**  RCT and crossover trials^a^  Pre-post trials | 0.36 (-0.82, 1.56) | 0.51 | 83.85% (<0.0001) |
|  | HFD duration (n=13) | 0.01 (-0.0007 0.03) | 0.06 | 78.44% (<0.0001) |
|  | Fat intake (n=8) | -0.003 (-0.007, 0.0006) | 0.08 | 58.92% (0.03) |
|  | Body mass (n=11) | 0.08 (-0.06, 0.22) | 0.23 | 80.48% (<0.0001) |
|  | **Physical activity status (n=13)**  Inactive^a^  Active | -0.72 (-1.38, -0.06) | 0.03* | 76.47% (<0.0001) |
|  | **Energy intake (n=13)**  Normocaloric diet^a^  Hyper-caloric diet | 0.56 (-0.14, 1.28) | 0.10 | 78.72% (<0.0001) |
|  | IMCL (n=13) | -0.44 (-1.29, 0.41) | 0.27 | 82.9% (<0.0001) |
| Circulating insulin | **Study design (n=12)**  RCT and crossover trials^a^  Pre-post trials | 0.02 (-0.94, 0.98) | 0.96 | 76% (<0.0001) |
|  | HFD duration (n=12) | 0.003 (-0.01, 0.02) | 0.70 | 75.99% (<0.0001) |
|  | Fat content (n=7) | -0.001 (-0.003, 0.002) | 0.47 | 0% (0.61) |
|  | Body mass (n=10) | 0.02 (-0.09, 0.14) | 0.64 | 73.31% (<0.0001) |
|  | **Physical activity status (n=12)**  Inactive^a^  Active | -0.58 (-1.08, -0.07) | 0.02* | 59.53% (0.007) |
|  | **Energy intake (n=12)**  Normocaloric diet^a^  Hyper-caloric diet | 0.85 (0.50, 1.19) | <0.01* | 15.49% (0.35) |
|  | IMCL (n=12) | 0.22 (-0.47, 0.93) | 0.48 | 74.98% (<0.0001) |
| Circulating glucose | **Study design (n=12)**  RCT and crossover trials^a^  Pre-post trials | 0.25 (-0.66, 1.18) | 0.54 | 72.33% (0.0007) |
|  | HFD duration (n=12) | 0.0006 (-0.01, 0.01) | 0.92 | 75.18% (0.0006) |
|  | Fat content (n=7) | -0.001 (-0.006, 0.004) | 0.71 | 76.01% (0.003) |
|  | Body mass (n=10) | 0.01 (-0.10, 0.14) | 0.76 | 74.99% (0.001) |
|  | **Physical activity status (n=12)**  Inactive^a^  Active | -0.29 (-0.87, 0.28) | 0.27 | 69.88% (0.004) |
|  | **Energy intake (n=12)**  Normocaloric diet^a^  Hyper-caloric diet | 0.31 (-0.28, 0.90) | 0.26 | 68.65% (0.005) |
|  | IMCL (n=12) | -0.04 (-0.74, 0.64) | 0.88 | 74.36% (0.0004) |
| HOMA-IR | HFD duration (n=4) | -0.004 (-0.03, 0.02) | 0.57 | 0% (0.54) |
|  | Fat content (n=3) | 0.002 (-0.03, 0.03) | 0.58 | 0% (0.45) |
|  | Body mass (n=4) | -0.02 (-0.21, 0.17) | 0.67 | 0% (0.49) |
|  | **Physical activity status (n=4)**  Inactive^a^  Active | 0.39 (-1.14, 1.93) | 0.38 | 0% (0.8) |
|  | **Energy intake (n=4)**  Normocaloric diet^a^  Hyper-caloric diet | 0.51 (0.07, 0.95) | 0.03* | 0% (0.64) |
|  | IMCL (n=4) | 0.43 (-1.03, 1.88) | 0.33 | 0% (0.96) |
| Hyperinsulinemic -euglycemic Clamp | **Study design (n=7)**  RCT and crossover trials^a^  Pre-post trials | 0.72 (-0.36, 1.79) | 0.15 | 0% (0.87) |
|  | HFD duration (n=7) | 0.002 (-0.006, 0.01) | 0.55 | 0% (0.49) |
|  | Fat content (n=4) | -0.001 (-0.008, 0.005) | 0.42 | 4.18% (0.30) |
|  | Body mass (n=5) | 0.02 (-0.06, 0.09) | 0.53 | 0% (0.82) |
|  | **Physical activity status (n=7)**  Inactive^a^  Active | -0.018 (-0.41, 0.37) | 0.91 | 0% (0.45) |
|  | **Energy intake (n=7)**  Normocaloric diet^a^  Hyper-caloric diet | -0.06 (-0.39, 0.36) | 0.92 | 0% (0.45) |
|  | IMCL (n=7) | -0.38 (-1.09, 0.34) | 0.23 | 0% (0.71) |

^a^Reference category in the model

Notes: 95% CI = 95% confidence interval; n, number of effect estimates; RCT, randomised controlled trial; χ^2^ = chi-squared test.
